# Development of a Validated LC-MS Method for the Determination of Cannabinoids and Evaluation of Supercritical CO_2_ vs. Ultrasound-Assisted Extraction in *Cannabis sativa* L. (Kompolti cv.)

**DOI:** 10.3390/antiox14070777

**Published:** 2025-06-24

**Authors:** Vasileios A. Ioannidis, Varvara Sygouni, Sotirios Giannopoulos, Konstantinos Sotirianos, Theophilos Ioannides, Christakis A. Paraskeva, Fotini N. Lamari

**Affiliations:** 1 Department of Pharmacy, University of Patras, GR-26504 Patras, Greece; ioannidis.v@ac.upatras.gr (V.A.I.); up1088246@ac.upatras.gr (S.G.); 2 Foundation for Research and Technology, Hellas, Institute of Chemical Engineering Sciences, FORTH/ICE-HT, GR-26504 Patras, Greece; theo@iceht.forth.gr (T.I.); takisp@chemeng.upatras.gr (C.A.P.); 3 Department of Chemical Engineering, University of Patras, GR-26504 Patras, Greece; konstantinos.sotirianos@gmail.com

**Keywords:** *Cannabis sativa* L., Kompolti, hemp, extraction, supercritical CO_2_, LC-MS, method validation, cannabinoids, antioxidant capacity

## Abstract

Cannabis (*Cannabis sativa* L.) contains numerous secondary metabolites with different bioactivities. Extraction methods differ in their efficiency in recovering metabolites from plant material, and thus cannabis extracts vary significantly in their composition and activity. We aimed to develop a repeatable and accurate HPLC-MS method for the determination of nine common cannabinoids and compare two widely used extraction techniques: ultrasound-assisted extraction (UAE) with methanol and supercritical CO_2_ extraction (SFE). Inflorescences of the Kompolti cultivar were used as the plant material. On a polar C18 column, more than thirty compounds were well separated within 25 min; thirteen cannabinoids were identified and eight of them were quantified, with cannabidiol and its acidic precursor being the most abundant. Additionally, three spectrophotometric assays were employed for extract characterization: the total phenolic content, total flavonoid content, and DPPH radical scavenging capacity. The SFE extract, obtained using ethanol as a co-solvent under low pressure (<100 bar) and temperature (<45 °C), was more enriched than the UAE extract (181.62 ± 2.90 vs. 140.64 ± 13.24 mg quercetin equivalents/g of dry extract) and cannabinoids (446.29 ± 22.66 vs. 379.85 ± 17.16 mg/g of dry extract), especially cannabinoid acids. However, UAE achieved greater recovery from the plant material (cannabinoids: 83.42 ± 5.15 vs. 68.84 ± 3.49 mg/g of plant material) and showed superior antioxidant capacity (DPPH IC_50_: 2.50 ± 0.18 vs. 3.37 ± 0.07 mg/mL). Notwithstanding the observed partial decarboxylation, the high repeatability (RSD < 15%, *n* = 11) of the entire analytical workflow involving UAE extraction and LC-MS analysis renders it suitable for routine analyses. This study contributes to the ongoing efforts toward the quality control and valorization of *C. sativa*.

## 1. Introduction

Cannabis (*Cannabis sativa* L.) is a medicinal plant that has been widely used not only for its fiber, but also for nutritional, recreational, religious, and therapeutic purposes [1]. Numerous studies have demonstrated the therapeutic potential of cannabis and cannabis-based medicines in the management of a wide range of diseases, mainly chronic pain, neurological and psychiatric disorders, cancer, and more [2,3,4,5,6]. Its therapeutic value is attributed to its rich profile of bioactive secondary metabolites [7,8,9,10,11]. Cannabis exhibits remarkable chemical diversity, with over 500 compounds identified to date including cannabinoids, terpenes, and phenolic compounds such as flavonoids, stilbenoids, and lignans [12,13,14].

Cannabinoids, the main group of secondary metabolites, constitute a distinct class of terpenophenolic compounds that are typically characterized by a C_21_ scaffold (C_22_ for the corresponding acids). They can be distinguished into 11 groups according to their structure: (-)-Δ^9^-*trans*-tetrahydrocannabinol (Δ^9^-THC), (-)-Δ^8^-*trans*-tetrahydrocannabinol (Δ^8^-THC), cannabigerol (CBG), cannabichromene (CBC), cannabidiol (CBD), cannabinodiol, cannabielsoin, cannabicyclol, cannabinol (CBN), cannabitriol, and miscellaneous compounds [12]. These metabolites are not evenly distributed in the plant but are abundant in the glandular trichomes of the female flowers [15]. Flavonoids and stilbenoids are primarily located in the aerial parts of the plant (leaves, flowers, stems), whereas lignans are found mainly in the roots, seeds, and fruits [16].

Cannabinoids naturally occur in their carboxylated forms and can be converted into the corresponding neutral compounds via decarboxylation (i.e., the removal of the carboxyl group at the C-2 position). This transformation typically occurs during plant drying and storage, or can be induced by heating [17,18]. In addition, cannabinoids may undergo further chemical transformations, including oxidation, isomerization, and photochemical reactions, triggered by environmental factors (e.g., oxidative stress, extreme temperatures, or UV radiation) or during post-harvest storage, leading to the formation of compounds such as Δ^8^-THC and CBN [12,17,18]. The cannabinoid profile of *Cannabis* plants depends on multiple factors including the variety and genotype, cultivation conditions, and harvest season [15].

Cannabis is considered to represent a single, highly diverse species—*Cannabis sativa* L.—in contrast to earlier classifications that proposed two or three distinct species within the genus (i.e., *C. sativa*, *C. indica*, and *C. ruderalis*) [12]. The intensive breeding and selection of *C. sativa* for specific traits have yielded a broad spectrum of cultivars with distinct phytochemical profiles [11]. Consequently, chemotypic (or chemovar) classification has emerged as a useful framework for differentiating varieties with diverse biological and pharmacological properties. In particular, five chemotypes are recognized based on their cannabinoid content: chemotype I or drug-type (THC:CBD ratio > 5.0, THC dominant); chemotype II or intermediate-type (THC:CBD ratio 0.2–5.0); chemotype III or fiber-type/hemp (THC:CBD ratio < 0.2, CBD dominant); chemotype IV (CBG dominant), and chemotype V (“zero cannabinoid” type) [19,20].

The comprehensive analysis of the rich and complex chemical composition of cannabis remains a significant analytical challenge. Many analytical techniques have been developed for the qualitative and quantitative analysis of *C. sativa* including TLC and HPTLC, NMR, FTIR, gas chromatography (GC-FID, GC-MS, GC × GC), and liquid chromatography (HPLC-UV/DAD, LC-MS, LC-MS/MS) [21,22]. Among these, gas and liquid chromatography are the most widely applied. GC allows for the simultaneous analysis of cannabinoids and terpenes in cannabis; however, since cannabinoid acids are heat-labile, they undergo decarboxylation upon sample introduction and analysis. To avoid their decomposition, derivatization of the sample prior to analysis is required [23]. On the other hand, liquid chromatography enables the separation of acidic and neutral cannabinoids without the need for any special sample pretreatment, facilitating routine analyses [21,22]. Mass spectrometry is considered superior to other detection methods for both qualitative and quantitative cannabis analysis, as it enables compound identification based on the molecular mass-to-charge ratio (*m*/*z*) of its ions. Currently, most methods for determining the cannabinoids in different matrices (biological fluids, food and feed products, plant materials) use tandem MS (MS/MS) detectors. These can resolve even co-eluting compounds, provided they differ slightly in their *m*/*z* values, thereby offering enhanced selectivity in complex sample matrices and unparalleled detection sensitivity [24,25,26]. Nevertheless, this equipment remains very expensive, and the selective ion monitoring techniques do not allow for the comprehensive monitoring of all cannabinoids and other metabolites, which is essential for proper quality control. Single MS detectors could address this need, but they suffer from narrow quantification limits. In contrast, UV detectors remain more attractive for routine quantitative analysis, especially for matrices with relatively high content of cannabinoids such as hemp [27].

Apart from the analytical technique employed, the choice of the extraction method is of critical importance, since it significantly influences the chemical composition, bioactivity, and physicochemical properties of the final extract [28,29]. Various extraction techniques have been reported for the recovery of cannabinoids and other compounds from cannabis including solvent-based and solventless extraction methods (ice-water extraction and mechanical pressing) [30,31,32,33]. Solvent-based techniques comprise Soxhlet extraction, dynamic maceration, ultrasound-assisted extraction (UAE), microwave-assisted extraction (MAE), pressurized liquid extraction (PLE), supercritical fluid extraction (SFE), and others [30,31,32,33]. Several solvents have been used including water (cannabis decoction/tea) [34], organic solvents (e.g., ethanol, methanol, acetonitrile, hexane, dichloromethane, chloroform) [31,35], vegetable oils (e.g., olive oil) [36,37,38], deep eutectic solvents [32,39], and supercritical and subcritical fluids, most often CO_2_ [40,41,42,43].

UAE is a commonly used extraction technique due to its high efficiency, low purchase and operating costs, and broad applicability at both the laboratory and industrial scales [32]. Ultrasound waves accelerate the extraction rate through the phenomenon of cavitation, which facilitates solvent penetration into the plant tissue and enhances mass transfer. As a result, UAE increases the extraction efficiency while significantly reducing the required time compared with conventional extraction methods [31,32]. On the other hand, SFE has emerged as a highly attractive technique for the recovery of valuable compounds from *C. sativa*, particularly cannabinoids. CO_2_ is the most frequently used supercritical fluid due to its relatively low critical point (T_c_: 31.1 °C and P_c_: 73.8 bar) [31,40]. Supercritical CO_2_ (scCO_2_) has several advantages over conventional organic solvents: it is neither toxic nor flammable, it has a low cost, it is recyclable, and it can be easily separated from the extract. scCO_2_ exhibits properties of both liquid and gas, and it can penetrate plant tissues more readily. Its physicochemical properties can be modified by simply changing the process parameters (temperature, pressure) [31,40]. scCO_2_ behaves as a non-polar solvent; by adding polar co-solvents, such as ethanol, methanol, and isopropanol, the selectivity of the extraction can be modified [41,42,43]. Finally, milder extraction conditions below the critical point can be used (subcritical CO_2_ extraction), targeting more volatile components of cannabis such as terpenes [40]. The unique combination of tunable solvent properties, environmental sustainability, and operational safety renders supercritical CO_2_ extraction a highly versatile and attractive technique, particularly in the pharmaceutical and food industries [32].

In the present study, emphasis was placed on the development and validation of a simple and reliable HPLC-ESI-MS method that ensured the background separation of as many cannabinoids as possible. We selected the more affordable HPLC-MS system (electrospray ionization—single quadrupole detector, ESI-SQ-MS) to develop a single chromatographic method for both the qualitative characterization and quantitative determination of cannabinoids in the plant samples. Moreover, the repeatability of cannabis extraction using UAE was assessed, with the goal of establishing a standardized protocol for routine analysis. Supercritical CO_2_ extraction, an environmentally sustainable alternative, was investigated and compared with UAE in terms of cannabinoid extractive efficiency. Three spectrophotometric assays—namely, the total phenolic content (TPC), total flavonoid content (TFC), and DPPH radical scavenging capacity—were additionally applied to the comprehensive chemical characterization of *C. sativa* extracts. We focused on the widely cultivated, CBD-dominant Kompolti variety, aiming to contribute to the existing knowledge of its phytochemical composition.

## 2. Materials and Methods

### 2.1. Chemicals and Reagents

All cannabinoid reference standards used, namely cannabidiol (CBD), Δ^9^-tetrahydrocannabinol (Δ^9^-THC), Δ^8^-tetrahydrocannabinol (Δ^8^-THC), cannabinol (CBN), cannabidivarinic acid (CBDVA), cannabidiolic acid (CBDA), cannabigerolic acid (CBGA), tetrahydrocannabivarinic acid (THCVA), tetrahydrocannabinolic acid (THCA), and cannabicromenic acid (CBCA) were of high purity (>99.0%). CBD (10 mg) was purchased from Phytolab (PhytoLab GmbH & Co. KG, Vestenbergsgreuth, Germany) as a crystal powder. Δ^9^-THC (1.0 mg/mL in ethanol) and Δ^8^-THC (1.0 mg/mL in ethanol) were purchased from Lipomed (Lipomed AG, Basel, Switzerland). CBN (1.0 mg/mL in methanol) was purchased from Supelco (Merck KGaA, Darmstadt, Germany). The rest of the cannabinoid acid standards were purchased as a mixed solution (0.5 mg/mL, each analyte, in 1% DIPEA and 0.05% ascorbic acid in acetonitrile) from Supelco (Merck KGaA, Darmstadt, Germany).

All solvents and buffers, namely, water, methanol, formic acid, and ammonium formate, were LC-MS grade, unless stated otherwise. These were obtained from Fisher Chemicals (Loughborough, UK), apart from ammonium formate, which was purchased from Sigma-Aldrich (Merck KGaA, Darmstadt, Germany). High-purity CO_2_ (99.99%) was used for the supercritical fluid extraction of cannabis samples, and ethanol 96% *v*/*v* was used as a co-solvent. The Folin–Ciocalteu reagent (for microscopy) and aluminum chloride hexahydrate (AlCl_3_ 6H_2_O, analytical grade) were purchased from Carlo Erba (Cornaredo, Italy); NaNO_2_ from LabKem (Dublin, Ireland); gallic acid (>98%) from Fluka (Buchs, Switzerland); Na_2_CO_3_ and NaOH (pellets, >99%) from Chem-Lab (Zedelgem, Belgium); ascorbic acid from Penta (Prague, Czech Republic); and quercetin from ChemCruz (Dallas, TX, USA).

### 2.2. Plant Material

Whole, air-dried female inflorescences of *Cannabis sativa* L. (Kompolti cv.) were used in the present study. The plant material (hemp) was cultivated outdoors by a registered producer in Magnesia, Greece, in 2021. The CBD content of the hemp flowers, according to the supplier, amounted to 7–8% (total CBD). The plant material was stored in airtight containers in a cool, dry, and dark environment until use in the experiments.

### 2.3. Extraction Methodology

#### 2.3.1. Ultrasound-Assisted Extraction (UAE)

Approximately 1 g of dried Cannabis flos, weighed on an analytical balance (Kern & Sohn GmbH, Balingen, Germany), was mildly shredded using a commercial blender and transferred to a round-bottom flask with 10 mL of methanol (1:10 plant material-to-solvent ratio, *w*/*v*). Then, the mixture was placed in an ultrasound bath (ultrasonic power: 120 W, frequency: 40 kHz, ISOLAB Laborgeräte GmbH, Eschau, Germany) for 30 min at ambient temperature (<40 °C) under reflux, to prevent solvent evaporation. After filtration, the plant material was returned to the round-bottom flask, and the process was repeated twice (in total, three times). The extracts were mixed and brought to dryness by removing methanol in a rotary evaporator (RotaVapor R-205, Büchi Labortechnik AG, Flawil, Switzerland). The dried extract was accurately weighed, and the extraction yield was calculated using the following equation:

yield% = (mass of dry extract/mass of extracted plant material) × 100%.(1)

Finally, the dried extract was stored at 4–6 °C in airtight containers, protected from light, and re-dissolved in methanol at the appropriate concentration prior to analysis.

#### 2.3.2. Supercritical CO_2_ Extraction (SFE)

**Figure 1:**
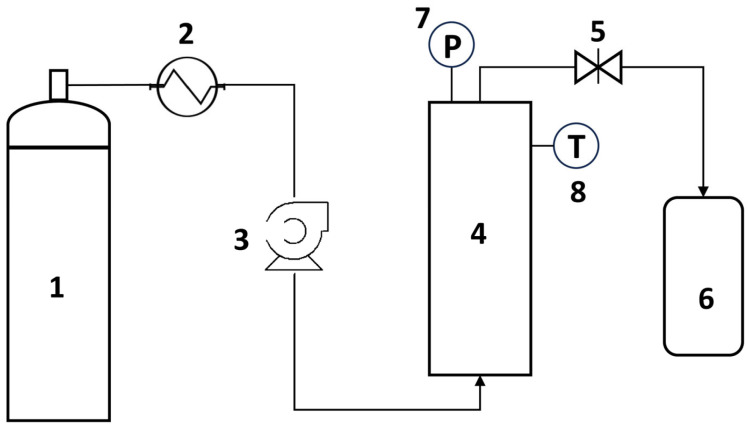
Schematic representation of the supercritical CO_2_ extraction apparatus: (1) CO_2_ cylinder; (2) CO_2_ cooler; (3) CO_2_ pump; (4) extraction vessel; (5) CO_2_ outlet control valve; (6) collector vessel; (7) manometer; (8) temperature indicator.

Supercritical CO_2_ extraction experiments were conducted in a cylindrical vessel of 450 mL made of stainless steel, equipped with appropriate fittings for hermetic sealing. The vessel was heated at the desired temperature with a heating mantle. For the injection of CO_2_, a supercritical constant flow pump (Supercritical 24 constant flow pump, Teledyne SSI) was employed. The flow rate of the pump was achieved with two standard 12 mL pump heads, which could be set in 0.01 mL/min increments from 0.00 to 24.00 mL/min. Pressure could be set in 10 psi increments from 0 to 10,000 psi. The pump head/heatsink was equipped with a cooler to pump the supercritical fluid into the vessel (to reduce the temperature of CO_2_ coming from the cylinder before pumping to avoid cavitation causes in the pump). The heating mantle, which covered the extraction vessel, was connected to a digital temperature controller. The vessel’s outlet was stainless-steel tubing (Premium Grade 304) that was 100 ft in length and had an outer diameter of 1/16 in and 0.010 in internal diameter. The outlet of the vessel was valve-controlled, and the length of the tubing was long enough to release the CO_2_ as gas. The vessel’s pressure could either be read on the pumping system device or the manometer connected to the stainless-steel vessel. The upper limit of pressure was set to 1500 psi (103.4 bar); thus, the pump decreased or stopped CO_2_ injection when 1650 psi was reached. The extract was collected in a vial at atmospheric pressure, which was placed in a thermostatic water bath at 25 °C. The overall scheme of the apparatus is presented in Figure 1.

Dried female Cannabis flos were used in the SFE experiments. The tested parameters included the extraction feed (hemp mass to be extracted, which varied between 5 and 30 g), the addition of ethanol as a co-solvent (1:3–1:6 feed-to-ethanol ratio, *w*/*v*), and the extraction time (4–12 h). Before extraction, the plant material was sieved to remove particles smaller than 180 μm. The co-solvent was added to the extraction vessel containing the plant material and briefly macerated before the extraction, since it was not possible to channel it through a second pump. The extraction process was conducted in 30-min cycles, where for 15 min the extraction was performed under stationary conditions (CO_2_ flow with closed outlet valve), followed by 15 min under dynamic conditions (CO_2_ flow with open outlet valve).

The duration of the extraction experiments ranged from 4 to 12 h. During the extraction process and throughout the parametric study, the temperature, pressure, and CO_2_ flow rate were kept within relatively steady ranges (T: 37–45 °C; P: 74–100 bar; flow rate: 3–5 mL/min).

The dried cannabis extracts were accurately weighed to calculate the extraction yield using Equation (1) and stored in airtight containers at 4–6 °C in the dark. The extracts were dissolved in methanol to the desired concentration before analysis.

### 2.4. Instrumentation and Chromatographic Conditions for LC-MS Analysis

All analyses were carried out on a 1260 Infinity II LC System (Agilent Technologies Inc., Santa Clara, CA, USA) that consisted of a multicolumn thermostat (G7116A, 1260 MCT), an autosampler (G7129A, 1260 Vial sampler), and a quaternary pump (G7111B, 1260 model) coupled to a single quadrupole (SQ) equipped with an electron spray ionization source (ESI) (G6125C, InfinityLab LC/MSD). The column used for the chromatographic separation was a Kinetex^®^ Polar C18 (100 × 3.0 mm i.d., 2.6 µm, 100 Å) (Phenomenex, Torrance, CA, USA).

During the development and optimization of the analytical method, different conditions and parameters were tested (mobile phase, gradient elution, buffers/acidic additives, oven temperature, flow rate). The method used by De Backer et al. [44] was modified to achieve the optimal resolution. The mobile phase consisted of H_2_O with 0.1% formic acid and 10 mM of ammonium formate (mobile phase A), and MeOH with 0.1% formic acid (mobile phase B). A simple gradient elution program was established: starting at 67% B and increased to 87% B in 25 min, held at 87% B for 3 min, then raised to 95% B in 1 min and held at 95% B for 5 min, decreased to 67% B in 1 min and held at 67% B for 5 min for re-equilibration of the column before the next injection, for a total run time of 40 min. The flow rate was set to 0.3 mL/min, the injection volume was 1 μL, the column oven temperature was kept constant at 30 °C, and the autosampler was thermostated at 10 °C. All samples were diluted in methanol and filtered through a PTFE syringe filter (pore size: 0.20 μm, filter diameter: 15 mm, Chromafil, Macherey-Nagel, Düren, Germany) before analysis. Each sample analysis was performed twice.

Mass spectrometry acquisition was performed using a single quadrupole. The ESI source was operated in both positive and negative ionization scan mode, *m*/*z* range: 150–900; scan time: 500 ms; fragmentor: 135 V; gain factor: 1. The MS source parameters were set as follows: drying gas (N_2_) temperature: 300 °C; drying gas (N_2_) flow rate: 11 L/min; nebulizing gas (N_2_) pressure: 15 psi; capillary voltage: 4000 V for both positive and negative ion mode.

The instrument was controlled by OpenLab CDS software (version 2.8). All data were collected in centroid mode, acquired using the same software.

### 2.5. LC-MS Quantification

Quantification of cannabinoids in the extracts was performed via the external standard method, using the reference standards described above. A stock solution of CBD (2.0 mg/mL) was prepared by dissolving the CBD crystal powder in methanol, followed by serial dilutions. Since the rest of the cannabinoid standards were supplied as solutions (1.0 mg/mL for Δ^9^-THC and CBN, 0.5 mg/mL for the cannabinoid acid mixture), serial dilutions were prepared. For CBD and Δ^9^-THC, five-point calibration curves were constructed: 100, 250, 333, 500, and 667 μg/mL; for CBN, a seven-point calibration curve was made: 5, 10, 50, 100, 250, 333, and 500 μg/mL. For the cannabinoid acids, four-point calibration curves were prepared: 50, 100, 250, and 333 μg/mL. Each concentration was analyzed twice and then averaged to construct the curves. In total, nine calibration curves were constructed, response peak area vs. the concentration, based on the peak area from the respective extracted ion chromatograms (EICs) in negative ionization mode: 313–314 *m*/*z* for CBD and Δ^9^-THC; 309–310 *m*/*z* for CBN; 357–358 *m*/*z* for CBDA, THCA, and CBCA; 359–360 *m*/*z* for CBGA; 329–330 *m*/*z* for CBDVA and THCVA. No calibration curve was constructed for Δ^8^-THC since the compound was not detected in any sample.

### 2.6. LC-MS Method Validation

The LC-MS method was validated in accordance with the ICH and EMA guidelines for the validation of the analytical procedures [45,46]. Method performance was evaluated in terms of specificity, linearity range, lower limit of detection (LLOD), lower limit of quantification (LLOQ), accuracy, and precision.

Selectivity (specificity) was assessed by reviewing the resolution (R_s_) between the least separated peaks in the chromatograms. R_s_ was determined as:

R_s_ = [t_R_(B) − t_R_(A)]/[0.5 × (W_A_ + W_B_)], (2)

where t_R_(A) and t_R_(B) are the retention times of compound A and B, respectively (t_R_(A) < t_R_(B)), W_A_ and W_B_ are the baseline widths of the chromatographic peaks A and B, in the same units as retention time. An R_s_ value greater than 1 was considered adequate.

The linearity range of the constructed calibration curves was estimated by evaluating the squared correlation coefficient (R^2^). A value greater than 0.99 was considered satisfactory. LLOD and LLOQ were determined as:

LLOD = 3.3 × *σ*/*S*, (3)

LLOQ = 10 × *σ*/*S*, (4)

where *σ* is the standard deviation (SD) of the response and *S* is the slope of the calibration curve of each cannabinoid.

The accuracy and precision of the analytical method were determined through the standard addition method (spike study). Different cannabis samples (extracts) were spiked with known amounts of standard compounds at three different concentration levels (*n* = 3 per level), covering the range of linearity of each calibration curve. Each cannabinoid was analyzed in triplicate at every concentration level, on three consecutive days, to assess the intra- and inter-day precision. Then, the % recovery of the cannabinoids was calculated to evaluate the accuracy. A recovery value between 85% and 115% (80–120% close to the LLOQ) was considered acceptable. Precision (intra-day and inter-day precision) was evaluated by measuring the relative standard deviation (RSD) of the concentration of each cannabinoid. An RSD value up to 15% (20% at a concentration close to the LLOQ) was considered acceptable.

### 2.7. Spectrophotometric Assays

All measurements for the spectrophotometric assays (i.e., total phenolic content (TPC), total flavonoid content (TFC), and DPPH radical scavenging capacity) were performed on a microplate reader (Sunrise^®^, Tecan, Männedorf, Switzerland). All experimental determinations were carried out in triplicate and expressed as the average ± SD, unless stated otherwise.

#### 2.7.1. Determination of Total Phenolic Content (TPC)

The determination of the total phenolic content of the cannabis extracts was carried out using the Folin–Ciocalteu method [47] with slight modifications. Briefly, 20 μL of the sample (extract or standard solution) in methanol (HPLC grade) was added to a 96-well microplate, followed by 180 μL of H_2_O (HPLC grade), 20 μL of 13.75% (*w*/*v*) Na_2_CO_3_, and 20 μL of Folin reagent (10%, *v*/*v*). The microplate was incubated for 30 min at ambient temperature in the absence of light. Then, the absorbance was measured at 750 nm against blank solutions. Gallic acid was used as a standard, and the results were expressed as mg of gallic acid equivalents (mg GAE) per mass of dry extract or plant material. A stock solution of 1 mg/mL of gallic acid in methanol was prepared, followed by serial dilutions to obtain seven standard solutions (i.e., 500, 250, 200, 150, 100, 75, 50 μg/mL), which were used to construct the calibration curve.

#### 2.7.2. Determination of Total Flavonoid Content (TFC)

The total flavonoid content of the cannabis extracts was determined using the aluminum chloride colorimetric assay [39] with some modifications. Shortly, 2.5 μL of the sample (extract or standard solution) in methanol (HPLC grade) and 7.5 μL of 5% (*w*/*v*) NaNO_2_ were added and mixed in a 96-well microplate. After 6 min of incubation, 15 μL of 10% (*w*/*v*) AlCl_3_.6H_2_O was added and mixed. After 5 min, 50 μL of 1 M NaOH was added, and the final volume was adjusted with 150 μL H_2_O (HPLC grade). The microplate was left for 20 min at ambient temperature in the dark. Afterward, the absorbance was recorded against blank solutions at 492 nm. Quercetin was used as a standard flavonoid, and the results were expressed as mg of quercetin equivalents (mg QE) per mass of dry extract or plant material. A stock solution of 1 mg/mL of quercetin in methanol was prepared and subsequently diluted to obtain seven standard solutions at concentrations of 400, 300, 200, 150, 100, 75, and 50 μg/mL, which were used to construct the calibration curve.

#### 2.7.3. Determination of DPPH Radical Scavenging Capacity

The antioxidant capacity of the cannabis extracts was evaluated by determining the scavenging capacity of the 1,1-diphenyl-2-picrylhydrazyl (DPPH) free radical [48] with slight modifications. Briefly, 20 μL of the sample (extract or standard solution) in methanol (HPLC grade) and 180 μL of 0.1 mM DPPH working solution in methanol were added to a 96-well microplate and kept for 30 min at ambient temperature, in the absence of light. A control sample was prepared following the same procedure, using the sample solvent instead of the extract or standard solution. Absorbance was measured at 540 nm against blank solutions. The DPPH radical scavenging capacity was calculated as percentage inhibition (%IC) using the equation:

%IC = [(A_control_ − A_sample_)/(A_control_)] × 100%, (5)

where A_control_ and A_sample_ are the absorbance of the control and the sample solution, respectively. Then, using GraphPad Prism 7.0 (GraphPad Software, Inc., San Diego, CA, USA), the %IC was plotted against the logarithm of the sample’s concentration, and the IC_50_ value was determined, denoting the sample concentration required to scavenge 50% of the DPPH radicals. Ascorbic acid was used as a positive control, with an IC_50_ value of 0.03 mg/mL.

### 2.8. Statistical Analysis

Statistical analysis of the experimental data was performed using Microsoft Excel. First, in order to detect outliers, Grubb’s test was performed with a 95% confidence interval (*p* < 0.05). The equality of variances was investigated using the F-test. Statistical significance among different samples was assessed using one-way analysis of variance (ANOVA) with 95% CI. When one-way ANOVA revealed significant differences based on *p* value (<0.05), Tukey’s honest significance difference (HSD) post hoc test was performed to determine the significance of differences between pairs of groups mean. The Student’s *t*-test was used with 95% CI (*p* < 0.05) for pairwise comparison to evaluate significant differences between the UAE and SFE extracts. Pearson correlation coefficients (r) and the corresponding *p*-values were also calculated using Microsoft Excel to assess the linear relationships between the DPPH IC_50_ values and measured chemical parameters. Statistical significance was determined using the *t*-distribution formula for correlation, and differences were considered significant at *p* < 0.05.

## 3. Results and Discussion

### 3.1. Identification of Cannabinoids via LC-MS

**Figure 2:**
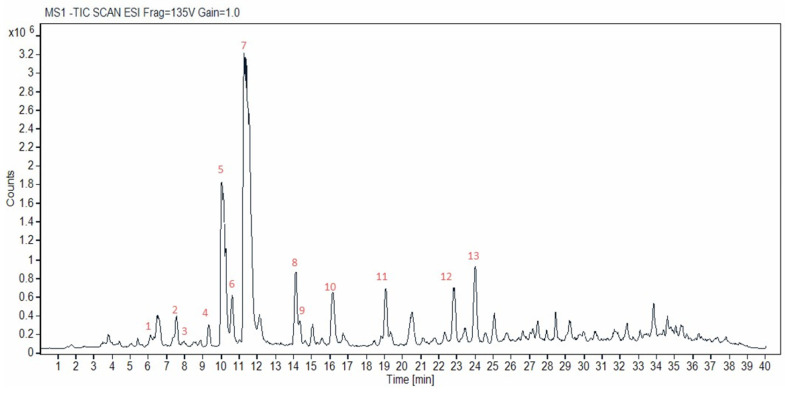
HPLC-MS total ion chromatogram of the scCO_2_ cannabis extract obtained in negative ionization mode. Peak numbering is in accordance with Table 1: CBDV (1), CBDVA (2), CBD-C4 (3), CBD-C4 (4), CBD (5), CBG (6), CBDA (7), CBGA (8), CBN (9), Δ^9^-THC (10), CBC (11), THCA (12). CBCA (13).

Identification of the major cannabinoids in the cannabis samples was performed using reference standards and by comparing chromatographic characteristics and mass spectral data with those in the literature [49,50,51,52,53]. Figure S1 shows the chromatograms of the reference standards used. A representative chromatogram of a supercritical CO_2_ cannabis extract acquired in negative ionization mode is shown in Figure 2. The identified cannabinoids are highlighted by numbers in the chromatogram and are listed in Table 1. Overall, over 30 peaks were separated in 25 min (40 min total run time), providing a well-resolved fingerprint of the studied hemp material, which could facilitate qualitative quality control. Thirteen were identified herein; however, more could be identified in the future, provided that more standards or purified compounds become available.

The compounds marked with an asterisk in Table 1 were confirmed using reference standards (i.e., CBDVA (2); CBD (5); CBDA (7); CBGA (8); CBN (9); Δ^9^-THC (10); THCA (12); CBCA (13)), thus their identification was considered certain. During the analysis of the mass spectra data, limited fragmentation of the compounds was observed, while in some cases, only the molecular ion was detected. This is to be expected, considering the use of a soft ionization source (ESI) and the gentle voltage applied. Furthermore, many cannabinoids showed identical fragments and were differentiated only by the relative intensities of the ions and their fragmentation patterns, as reported in the literature [50].

In the present study, a CBD-dominant cannabis plant was used (Kompolti cv.); thus, it was expected that cannabinoids belonging to the CBD group would be more abundant than other cannabinoid subclasses. For instance, the cannabinoid THCVA, for which a reference standard was available, was not found in any extract, nor were other minor cannabinoids of the Δ^9^-THC group.

Many cannabinoids have the same molecular mass. For example, CBD, Δ^9^-THC, Δ^8^-THC, CBC, and CBL show a mass of approximately 314 amu and their corresponding acids 358 amu. Other cannabinoid analogues show a similar pattern regarding their masses, with corresponding mass shifts that reflect specific structural modifications. Therefore, their identification can be achieved either using analytical standards or based on the elution order, fragmentation pattern, and the relative peak intensities in the mass spectra. On the other hand, CBG is easily distinguished from the rest of the cannabinoids, as it shows a molecular mass of 316 amu. In our case, CBG (6) and its corresponding acid, CBGA (8), eluted at 10.76 min and 14.76 min, exhibited the precursor ions 317[M+H]^+^ *m*/*z* and 361[M+H]^+^ *m*/*z*, respectively. Likewise, CBN (9) with a distinct molecular ion of 311[M+H]^+^ *m*/*z* eluted at 14.47 min.

Under the chromatographic conditions used, neutral cannabinoids eluted earlier than their corresponding acidic forms. For example, the retention time of CBD was 10.29 min compared with 11.47 min for CBDA; similarly, Δ^9^-THC eluted at 16.28 min, while THCA at 22.84 min. Additionally, cannabinoids with a pentyl side chain (e.g., CBDA, THCA) eluted later than their corresponding propyl analogues (CBDVA, THCVA) (Figure S1). Thus, the elution time of cannabinoids for which no analytical standards were available could be predicted as previously mentioned by Berman et al. [50]. Within the same cannabinoid class, the retention time increases with the length of the aliphatic side chain. This was the case with CBDV (1), which eluted at 6.13 min, before CBDVA (2) and CBD (5). Consequently, the less abundant butyl-analogues were expected between the propyl- and pentyl-analogues, and so on. CBD-C4 (3) (MW: 300 amu), eluted at 7.99 min, between CBDV and CBD. Respectively, CBDA-C4 (4) eluted at 9.19 min, between CBDVA (2) and CBDA (7), and after its corresponding neutral cannabinoid CBD-C4 (3). Finally, CBCA (13) eluted after THCA (12), therefore, CBC (11) is expected to elute after Δ^9^-THC (10) and before CBCA (13). The only peak in this time interval with a molecular ion 315 *m*/*z* [M+H]^+^ eluted at 19.19 min, which was attributed to CBC (11).

Regarding the acidic cannabinoids, analysis of the mass spectra in the positive ionization mode revealed the frequent formation of the positively charged adducts [M+Na]^+^ and [M+H–H_2_O]^+^, which in conjunction with the molecular ion [M+H]^+^, create a characteristic pattern that facilitates the elucidation of the molecular mass of unknown compounds and distinguishing acidic from neutral cannabinoids. Although the [M+Na]^+^ conjugate was also observed in neutral cannabinoids, its abundance was considerably lower. Furthermore, acidic cannabinoids, in addition to the loss of 18 amu (-H_2_O group), typically showed a loss of 44 amu, attributed to the cleavage of the carboxyl group they bear, yielding the product ion [M+H–CO_2_]^+^.

### 3.2. LC-MS Method Performance Characteristics

#### 3.2.1. Linearity, LLOD, LLOQ, Specificity

In total, nine calibration curves were constructed using the reference standards as described in Section 2.5. For each compound (namely CBDVA, CBDA, CBGA, THCVA, THCA, CBCA, CBD, CBN, and Δ^9^-THC), the LLOD and LLOQ values, the linear regression equation, the linearity range, and coefficient of determination (R^2^) are listed in Table 2.

The selectivity of the method was evaluated by inspecting the chromatographic separation of the peaks of the main cannabinoid compounds. A satisfactory resolution (R_s_) > 1 was achieved for all compounds of interest. For example, the R_s_ values for the least separated peaks of Δ^9^-THC—THCVA and CBGA—CBN in the cannabis extract were 1.2 and 1.1, respectively. Although the separation of these peaks is not ideal, it is considered sufficient for their identification and quantification. In addition, the use of the mass spectrometer as a detector allows for the identification and quantification of peaks that are co-eluted (herein with EIC), as long as their molecular weight differs, as in the case of CBGA and CBN (360 and 310, respectively). In this method, we sacrificed the analysis time (compared with the <10-min run time typical of many chromatographic methods) and detection limits (μg/mL versus ng/mL in LC-MS/MS and low μg/mL in LC-UV methods) in order to achieve both good selectivity and reliable quantification using HPLC-MS [25]. A greater linearity range and lower quantification limits could be achieved if our separation system employed a UV detector, or if selected ion monitoring (SIM) mode were used for quantification in the MS detection.

#### 3.2.2. Spike Study

The accuracy of the method was determined by spiking a known amount of a reference standard compound to a sample and measuring the mean recovery at three concentration levels (*n* = 3 at each level), covering the linearity range of the calibration curve. Recovery values of Δ^9^-THC and CBN were assessed using the extract of the CBD-dominant variety, while that of CBD was evaluated using a THC-dominant variety. In either case, cannabis flowers were extracted with methanol via UAE. The simultaneous evaluation of the accuracy (and precision) of the analytical method for all cannabinoid acids was impossible, as the concentration of those compounds had significant variations within the same samples (e.g., the concentration of CBDA was multiple times that of CBGA, and the respective reference analytical standards were available as a mixed solution). Thus, extracts of decarboxylated THC-dominant cannabis plants were used to facilitate the study and reduce the number of analyses performed. More specifically, cannabis flowers were decarboxylated in an oven at 145 °C before the extraction, until no cannabinoid acids were detected. As shown in Table S1, heating of the plant material at 145 °C for 30 min was enough for the complete decarboxylation of cannabinoid acids, which is consistent with previous studies showing nearly complete decarboxylation at 140 °C after 30 min [18].

The recovery of the tested cannabinoids (CBDVA, CBDA, CBGA, THCVA, THCA, CBCA, CBD, CBN, and Δ^9^-THC) (Table S2) ranged between 92.87 and 109.27% in the mid-concentration levels, 88.10–106.98% in the higher levels, and 90.84–118.02% in the lower levels (close to LLOQ). In the case of CBD, the recovery (77.93–80.61%) at the higher level exceeded the desired limits of 85–115%, indicating increased uncertainty in the higher limits of the calibration curve and confirming the narrow quantification range of LC-MS [27]. Thus, the quantification of CBD was performed by injecting samples that fell at the middle levels of the calibration curve.

Intra-day and inter-day precision of the analytical method were evaluated by performing the analysis over three consecutive days, with three replicates per day, and calculating the RSD of the measured concentrations for each compound. The results are shown in Table S3. In all cases, the RSD was lower than 6% for repeatability and lower than 8% for intermediate precision, well within the 15% acceptance limit, highlighting the high precision of the analytical method.

### 3.3. Ultrasound-Assisted Extraction (UAE)

A comprehensive protocol for the routine determination of cannabinoids in plant samples requires a clearly defined extraction procedure. In our study, we employed ultrasound-assisted extraction (UAE) with methanol, as supported by several previous reports [36,54,55,56,57,58,59]. Notably, Tzimas et al. [35] identified methanol as the most effective solvent for cannabinoid recovery, following a systematic evaluation of fourteen solvents employing UAE. A total of 11 UAE experiments were conducted and analyzed via LC-MS. The repeatability of the entire process—including pre-treatment, extraction, and LC-MS analysis of the plant material—was assessed by comparing both the extraction yields and quantitative results. Furthermore, the total phenolic content (TPC) and total flavonoid content (TFC) were determined, and the antioxidant capacity of the extracts was also evaluated.

The mean extraction yield was 21.95 ± 0.61%, RSD: 2.79% (*n* = 11). The quantitative results of the LC-MS analysis of the UAE extracts are presented in Table S4, expressed as mg per g of plant material. For all cannabinoids, the RSD of the concentrations was lower than 15%. These results highlight the high repeatability of the entire process and suggest that UAE with methanol could be used for routine plant sample analysis.

The average CBD and CBDA content of the hemp “Kompolti cv.” flowers was 41.29 ± 2.73 mg/g and 35.62 ± 2.43 mg/g of plant material, respectively, whereas the Δ^9^-THC was 2.01 ± 0.17 mg/g. The THC:CBD ratio was calculated as 0.03, well below 0.2. The total cannabinoid (CNB) content was 83.42 ± 5.15 mg/g of plant material and calculated as the sum of all quantified cannabinoids via LC-MS. THCA and CBN, although detected in the extracts, were below the LLOQ, and therefore not quantified. CBG was not quantified, since the corresponding analytical standard was not available. THCVA was not detected in any extract. Finally, the TPC was 39.76 ± 4.36 mg GAE/g of plant material, while the TFC was 30.58 ± 2.89 mg QE/g of plant material. The antioxidant capacity of the extracts was expressed as the DPPH radical scavenging capacity, and an IC_50_ of 2.50 ± 0.18 mg of dry extract per mL of methanol was calculated.

The results of the UAE extraction of hemp flowers (Kompolti cv.) are consistent with the information provided by the supplier; according to the producer, hemp’s CBD content amounted to 70–80 mg/g (7–8% *w*/*w* Total CBD), while in our study, it was calculated at 72.53 ± 4.47 mg/g. Additionally, Spano et al. [58] studied the cannabinoid content of hemp flowers from the Kompolti cv. in three different harvest stages, from the beginning to the end of the plants’ flowering period. The researchers also used the UAE technique and methanol as an extraction solvent for the recovery of cannabinoids, and quantitative analysis was performed via UHPLC-DAD. They reported a substantially lower CBDA content compared with our study, ranging from 14.00 to 15.50 mg/g. However, regarding the CBD and Δ^9^-THC content, our results are consistent, falling within the ranges reported by the researchers (CBD: 29.00–50.90 mg/g and Δ^9^-THC: 1.40–3.00 mg/g). Additionally, in both studies, the THC:CBD ratio was lower than 1.0, while the THCA, CBN, and Δ^8^-THC could not be quantified. Finally, the TPC was determined using the Folin–Ciocalteu method and ranged between 2.11 and 2.57 mg GAE/g of fresh plant material, significantly lower compared with our study, even though the researchers utilized UAE and the MeOH/H_2_O 7:3 ratio (*v*/*v*) as an extraction solvent, targeting more specifically the recovery of phenolic compounds [58].

### 3.4. Supercritical CO_2_ Extraction (SFE) Results

A series of supercritical CO_2_ extractions was performed to investigate and improve the extraction process, in terms of extraction yield, chemical composition, and antioxidant capacity of the obtained extract. In particular, the effects of extraction feed (5–30 g), extraction time (4–12 h), and the addition of ethanol as a co-solvent in various ratios (1:3–1:6 feed-to-ethanol ratio, *w*/*v*) were studied. The temperature (T: 37–45 °C), pressure (P: 74–100 bar), and CO_2_ flow rate (3–5 mL/min) were kept relatively constant throughout the experiments. The extraction conditions applied, along with the respective yields, and the chemical composition of each extract (total CNBs, TPC, TFC, and DPPH IC_50_ values) are summarized in Table 3 and Figures S2 and S3.

The qualitative analysis did not show any noteworthy differences among the obtained extracts in terms of cannabinoids detected. On the other hand, quantitative analysis revealed some distinct differences. The results are summarized in Table 4, expressed as mg per g of dry extract and mg per g of plant material. The total CNB content (sum of quantifiable cannabinoids), total CBD, and total THC are also reported. THCVA was not detected in any extract. CBG, although detected, was not quantified since the respective reference analytical standard was not available. THC:CBD ratio was below 0.2 in all cases. Extracts 1 and 2 were not analyzed because the amount of the collected extract was insufficient (Table 3).

#### 3.4.1. Effect of Extraction Feed

A series of supercritical fluid extraction (SFE) experiments was conducted to determine the optimal extraction feed for the specific supercritical CO_2_ apparatus intended for subsequent steps. It has already been well-established that a decrease in the feed-to-solvent ratio leads to an enhancement in extraction efficiency [42]. Our results confirmed that decreasing the amount of hemp in the feed without changing the CO_2_ flow rate and extraction time (i.e., the amount of solvent consumed), significantly improved the extraction efficiency (Table 3). Specifically, when the vessel was fully packed (~30.0 g of hemp), the yield was low (0.15% *w*/*w*). Reducing the feed mass to 10.0 g increased the yield to 0.31% *w*/*w*. A further decrease in the feed to 4.7 g, corresponding to approximately 1:10 feed-to-extraction vessel ratio (*w*/*v*), improved the extraction yield, reaching 2.74% *w*/*w*. Additionally, as the extraction feed decreased, a concurrent and significant (*p* < 0.01) increase in the TPC, TFC, and antioxidant capacity of the extracts was observed.

#### 3.4.2. Effect of Ethanol Addition as a Co-Solvent

Previous studies by Gallo-Molina et al. [41] and Rovetto and Aieta [42] have reported that the addition of a small amount of ethanol as a co-solvent enhanced the extraction yield while reducing the extraction time. In line with these findings, the maximum yield was observed at a ratio of 1:3 of hemp-to-ethanol (*w*/*v*). As shown in Table 3, a small addition of ethanol greatly increased the extraction efficiency, with the yield increasing threefold from 2.74% (no. 3, feed: approx. 5 g) to 8.38% (no. 4, feed: 5 g). However, the further addition of co-solvent negatively affected the extraction yield, which tended to decline from 8.38% (no. 4, ratio: 1:3) to 7.59% (no. 6, ratio: 1:5) and 6.04% (no. 5, ratio: 1:6).

Moreover, the addition of ethanol significantly altered the chemical composition of the obtained extracts, even though the recovery of cannabinoids, flavonoids, and phenolics increased; CNBs: from 20.17 to 24.13–34.78 mg/g of plant material; TFC: from 2.26 to 9.50–14.05 mg QE/g of plant material; TPC: from 6.23 to 12.42–17.51 mg GAE/g of plant material (Table 3). At the same time, the selectivity of the extraction decreased. In the presence of ethanol, various compounds were co-extracted, as indicated by the extract’s color, which changed from orange to green, suggesting the co-extraction of chlorophylls and other impurities [41,42]. In particular, while the TFC significantly increased, a decrease in total cannabinoid content and antioxidant capacity (increase in IC_50_ value from 2.03 to 2.92–4.71 mg/mL) was observed compared with extracts obtained without the addition of co-solvent. Changes regarding the TPC of the extracts were insignificant (Table 3). Additionally, it was observed that the presence of ethanol decreased the recovery of CBD but increased that of all the acidic forms, particularly CBDVA, CBGA, CBCA, and THCA (Table 4), which is rational since ethanol’s addition increased the polarity of the solvent [42]. The chemical composition of the extracts was significantly affected by the presence of the co-solvent, but not by its quantity, since with an increase in the ethanol added, the observed discrepancies of the TPC, TFC, and total CNBs were insignificant (Table 3).

#### 3.4.3. Effect of Extraction Time

During experiment no. 7 (Table 3), the plant material was extracted for a total time of 12 h, while the obtained extract was collected in 4 h intervals, separately. A period of 8 h was enough to obtain most of the cannabis extract. Between time intervals 0–4 h (no. 7.1) and 4–8 h (no. 7.2), approximately the same amount of extract was collected, 0.394 g (yield: 7.82%) and 0.332 g (yield: 6.58%), respectively. After the 8-h timepoint (i.e., time interval 8–12 h (no. 7.3)), the extraction yield was 1.02% (Table 3).

The extraction time did not significantly affect the chemical composition of the extract (TPC, TFC, and total CNBs) during the first 8 h (Table 3 and Table 4). After the 8-h timepoint (extract no. 7.3), the total CNBs were increased (mostly because of the higher CBD content), while no difference was observed in the TPC and TFC. In addition, extract no. 7.3 showed a significantly greater antioxidant capacity compared with extracts no. 7.1 and 7.2, which can be attributed to the higher cannabinoid content.

### 3.5. Comparison Between UAE and SFE

**Figure 3:**
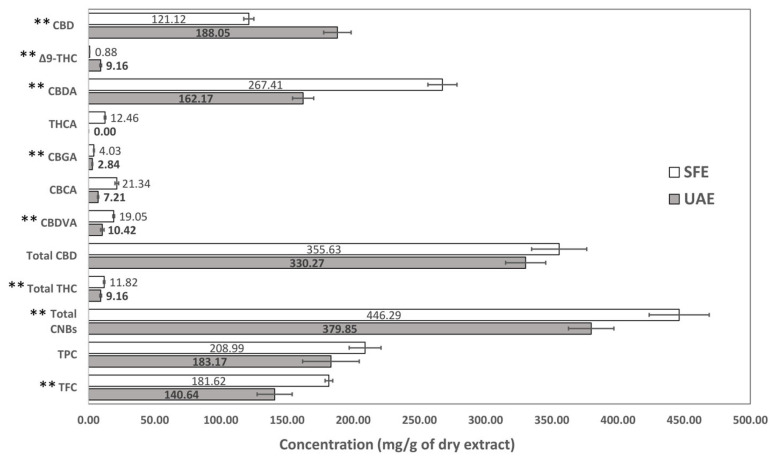
Comparison of cannabinoid content, TPC, and TFC of the UAE and SFE extracts (no. 7). The bar graph depicts the mean values, expressed as mg/g of dry extract ± SD. Asterisks indicate statistical significance for pairwise comparisons (** *p* < 0.01).

**Figure 4:**
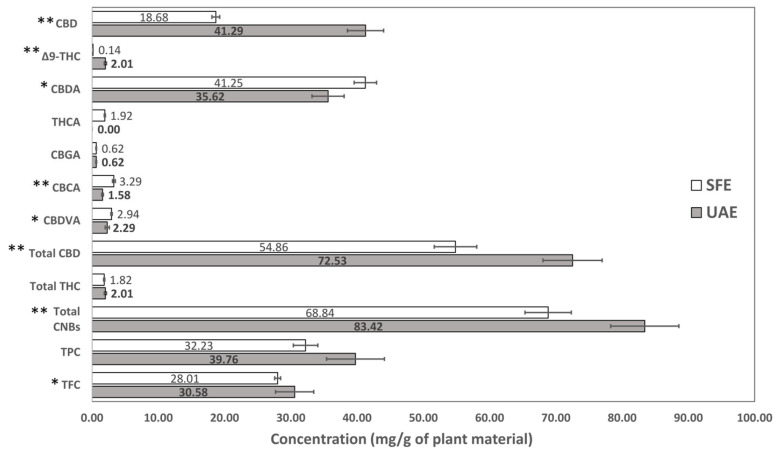
Comparison of cannabinoids, TPC, and TFC recovery via UAE and SFE (no. 7). The bar graph depicts the mean values, expressed as mg/g of plant material ± SD. Asterisks indicate statistical significance for pairwise comparisons (* *p* < 0.05; ** *p* < 0.01).

Ultrasound-assisted extraction (UAE) and supercritical CO_2_ extraction (SFE) are two widely used techniques for the recovery of cannabinoids. They differ in several key aspects including their underlying principles, equipment cost and availability as well as the nature of the extraction solvent (e.g., polarity, toxicity). A comparison of these two techniques in terms of chemical composition and the antioxidant capacity of the obtained extracts (Figure 3) and the recovery of bioactive compounds from cannabis flowers (Figure 4) was conducted in this study. In the case of UAE, methanol was used as a solvent (UAE extract, Section 3.3), while for the SFE, the best performing extract was selected (SFE extract, Section 3.4, extract no. 7; Table 3), which was obtained via scCO_2_ with the addition of ethanol as a co-solvent.

First and foremost, the extraction yield was 21.95 ± 0.61% (*w*/*w*) for the UAE and 15.42 ± 0.23% (*w*/*w*) for the SFE. By comparing the TPC, TFC, and total CNB content, it was ascertained that the SFE extract was more abundant in flavonoids and cannabinoids than the UAE extract (Figure 4). No significant TPC difference was observed between the two extracts. More specifically, in the SFE extract, although CBD and Δ^9^-THC were significantly lower than in the UAE extract (Figure 3), the content of all cannabinoid acids was higher. It is worth mentioning that THCA was detected but not quantified in the UAE extract, while in the SFE extract, it was 12.46 ± 0.59 mg/g (Figure 3). Furthermore, CBN was detected in both extracts but could not be quantified in either. Finally, the THC:CBD ratio was calculated equal to 0.03 (less than 0.2) in both cases, indicating consistent cannabinoid profiles. Considering the overall higher content of cannabinoid acids in the SFE extract, the significantly lower content of CBD as well as the similar total CBD, it is possible that during the UAE process, partial decarboxylation of the cannabinoid acids took place.

By comparing the two techniques in terms of recovery from cannabis flowers, it was observed that via UAE, more bioactive compounds were obtained per mass of plant material (Figure 4). However, the recovery of all cannabinoid acids was still higher in the case of SFE, except for CBGA, supporting the hypothesis of the partial decarboxylation of cannabinoid acids during UAE.

Rovetto and Aieta [42] reported that the content of cannabinoid acids in the SFE extracts was greatly influenced by pressure; the lower the pressure, the higher the content of acids. In the present study, during SFE extraction, low pressure was employed (<100 bar), which, along with the low temperature (up to 45 °C) and the presence of ethanol that better solubilizes them, preserved the natural acidic forms. In contrast, the UAE conditions may have promoted partial decarboxylation, resulting in lower levels of acidic cannabinoids—an effect not typically observed when UAE is compared to SFE under harsher parameters [41]. Cannabinoid acids are unstable, less potent, and less efficacious than their neutral forms (THC, CBD) at the CB_1_ and CB_2_ receptors, both in binding affinity and functional activity [43], underscoring the importance of thorough phytochemical characterization and the careful selection of both extraction and post-extraction conditions.

It is worth mentioning that the UAE extract had a significantly lower DPPH IC_50_ value than the SFE extract (UAE: 2.50 ± 0.18 mg/mL vs. SFE: 3.37 ± 0.07 mg/mL, *p*-value < 0.01), even though the latter was more abundant in bioactive compounds such as cannabinoids and flavonoids. The higher radical scavenging activity of the UAE extract may be attributed to the co-extraction of more redox-active compounds (e.g., chlorophylls), suggested by the notably higher extraction yield. In addition, the lower antioxidant capacity of the SFE extract could be linked to its higher content of acidic cannabinoids.

As previously reported by Dawidowicz et al. [60], cannabinoid acids possess lower DPPH antioxidant capacity than their neutral counterparts. This correlation was also verified in our study through the correlation analysis of the DPPH IC_50_ values with cannabinoid composition presented in Table 3 and Table 4. In particular, the total CNBs, Δ^9^-THC, and CBD showed moderate to strong negative correlations with the IC50 values (*r* = −0.743, −0.806, −0.686, respectively; *p* < 0.05), indicating that higher concentrations of neutral cannabinoids enhance antioxidant potential. In contrast, CBCA had a positive correlation with the IC_50_ values (*r* = 0.694, *p* < 0.05), suggesting that it may reduce radical scavenging activity. Moderate positive but not significant correlations were noted for all other acids and their sum. These results show that the cannabinoid content, and specifically the concentration of the neutral ones, is a major determinant of antioxidant capacity, whereas the acidic forms contribute less to antioxidant potential under DPPH conditions. The DPPH assay is based on the electron transfer mechanism, although hydrogen atom transfer can also contribute, and was selected it because it is better suited for moderately polar/lipophilic substrates [61]. These mechanistic considerations, along with the structural features of cannabinoid acids (electron-withdrawing carboxylic group, which reduces the ability of their phenolic groups to donate electrons or hydrogen atoms), likely explain their diminished performance in this assay. The employment of complementary assays, like ABTS and FRAP, could overcome the mechanistic limitations of the DPPH assay and provide a more comprehensive assessment of cannabis extract antioxidant capacity [60]. Nonetheless, in vivo activity remains far more complex due to factors such as bioavailability, metabolism, and pleiotropic effects.

## 4. Conclusions

In the present study, a simple and reliable LC-MS method for the quantitative determination of nine cannabinoids was developed and validated. Accuracy ranged from 88.1% to 109.3% (80.8% to 118.0% close to the LLOQ); only CBD showcased poorer recovery at the upper part of the calibration curve. High precision of the analytical method was achieved; intra-day RSD < 6%, and inter-day RSD < 8%. This LC-MS method was applied to the comprehensive characterization of major cannabinoids in the Kompolti hemp variety. In total, 13 cannabinoids were identified, and 8 were quantified, with CBD and CBDA being the predominant ones. It is worth mentioning that this method ensures the sufficient background separation of many cannabinoids (>30 in 25 min), and thus more cannabinoids could be determined with simple HPLC-MS or HPLC-UV, if the corresponding reference analytical standards were available. The high repeatability of the entire process, namely pre-treatment, extraction via UAE, and LC-MS quantitative analysis, was confirmed by the RSD, which did not exceed 15% in any case (*n* = 11).

Supercritical CO_2_ extraction (SFE) was investigated and compared with ultrasound-assisted extraction (UAE) in terms of cannabinoid extractive capacity. The extraction feed reduction and the addition of ethanol greatly enhanced the SFE process yield and the recovery of bioactive compounds. The relatively low pressure and temperature during SFE, along with the use of ethanol as a co-solvent, preserved the acidic cannabinoids, which seemed to be partially decarboxylated during UAE. The SFE extract exhibited comparable TPC, was richer in flavonoids and cannabinoids, but showed a lower overall recovery of bioactive compounds and reduced DPPH radical scavenging capacity.

Between the two techniques, UAE is less complex and enables the adequate recovery of cannabinoids. As such, it constitutes a reliable method for routine analysis, despite its tendency to promote partial decarboxylation. On the other hand, SFE involves higher costs and requires specialized personnel and equipment, but it offers advantages in applications where purity is preferred over absolute yield.

## Figures and Tables

**Table 1:** Identified cannabinoids in the hemp Kompolti cultivar extracts. For each compound, the retention time, name and molecular formula, molecular weight, and mass spectral data are provided. The mass spectral data include the main *m*/*z* peaks acquired by ESI-SQ-MS in both positive and negative ionization modes, showcasing the adducts, precursor ions, and product ions.

No.	Retention Time (min)	Compound	MW	Mass Spectra	
				*m*/*z* (+)	*m*/*z* (−)
1	6.13	Cannabidivarin CBDV (C_19_H_26_O_2_)	286	287[M+H]^+^ 219[C_14_H_18_O_2_+H]^+^ 165[C_10_H_12_O_2_+H]^+^	285[M–H]^−^ 217[C_14_H_18_O_2_–H]^−^
2	7.55	Cannabidivarinic acid CBDVA * (C_20_H_26_O_4_)	330	331[M+H]^+^ 313[M+H–H_2_O]^+^ 353[M+Na]^+^ 219[C_14_H_18_O_2_+H]^+^	329[M–H]^−^ 311[C_20_H_24_O_3_–H]^−^ 285[M–CO_2_H]^−^ 681[2M–H+Na]^−^
3	7.99	Cannabidiol-C4 (Cannabidibutol) CBD-C4 (C_20_H_28_O_2_)	300	301[M+H]^+^	299[M–H]^−^ 231[C_15_H_20_O_2_–H]^−^
4	9.19	Cannabidiolic acid-C4 (Cannabidibutolic acid) CBDA-C4 (CBDBA) (C_21_H_28_O_4_)	344	345[M+H]^+^	343[M–H]^−^ 709[2M–H+Na]^−^ 231[C_15_H_20_O_2_–H]^−^ 325[C_21_H_26_O_3_–H]^−^
5	10.29	Cannabidiol CBD * (C_21_H_30_O_2_)	314	315[M+H]^+^ 247[C_16_H_22_O_2_+H]^+^ 181[C_11_H_16_O_2_+H]^+ ^ 193[C_12_H_16_O_2_+H]^+^ 259[C_17_H_22_O_2_+H]^+^	313[M–H]^−^ 627[2M–H]^−^ 245[C_16_H_22_O_2_–H]^−^
6	10.76	Cannabigerol CBG (C_21_H_32_O_2_)	316	317[M+H]^+^ 193[C_12_H_16_O_2_+H]^+^	315[M–H]^−^
7	11.47	Cannabidiolic acid CBDA * (C_22_H_30_O_4_)	358	359[M+H]^+^ 341[M+H–H_2_O]^+^ 381[M+Na]^+^ 315[M+H–CO_2_]^+^ 219[C_13_H_14_O_3_+H]^+^	357[M–H]^−^ 737[2M–H+Na]^− ^ 313[M–CO_2_H]^−^ 245[C_16_H_22_O_2_–H]^− ^ 179[C_11_H_16_O_2_–H]^−^ 339[C_22_H_28_O_3_–H]^−^
8	14.19	Cannabigerolic acid CBGA * (C_22_H_32_O_4_)	360	361[M+H]^+^ 343[M+H–H_2_O]^+^ 383[M+Na]^+^	359[M–H]^−^ 741[2M–H+Na]^−^
9	14.47	Cannabinol CBN * (C_21_H_26_O_2_)	310	311[M+H]^+^ 241[C_16_H_16_O_2_+H]^+^	309[M–H]^−^
10	16.28	(-)-*trans*-Δ^9^-tetrahydrocannabinol Δ^9^-THC * (C_21_H_30_O_2_)	314	315[M+H]^+^ 193[C_12_H_16_O_2_+H]^+^ 247[C_16_H_22_O_2_+H]^+^ 181[C_11_H_16_O_2_+H]^+^ 259[C_17_H_22_O_2_+H]^+^	313[M–H]^−^ 627[2M–H]^−^
11	19.19	Cannabichromene CBC (C_21_H_30_O_2_)	314	315[M+H]^+^ 193[C_12_H_16_O_2_+H]^+^ 247[C_16_H_22_O_2_+H]^+^ 259[C_17_H_22_O_2_+H]^+^	313[M–H]^−^
12	22.84	Tetrahydrocannabinolic acid THCA * (C_22_H_30_O_4_)	358	359[M+H]^+^ 341[M+H-H_2_O]^+^ 381[M+Na]^+^ 315[M+H–CO_2_]^+^ 285[C_18_H_20_O_3_+H]^+^	357[M–H]^−^ 737[2M–H+Na]^−^ 313[M–CO_2_H]^−^
13	23.94	Cannabichromenic acid CBCA * (C_22_H_30_O_4_)	358	359[M+H]^+^ 341[M+H–H_2_O]^+^ 381[M+Na]^+^ 315[M+H–CO_2_]^+^	357[M–H]^−^ 737[2M–H+Na]^−^ 313[M–CO_2_H]^−^ 243[C_16_H_20_O_2_–H]^−^

* Compounds identified and confirmed using reference standards.

**Table 2:** Calibration curves of CBDVA, CBDA, CBGA, THCVA, THCA, CBCA, CBD, CBN, and Δ^9^-THC. The linearity range, LLOD, LLOQ, linear regression equation, and coefficient of determination (R^2^) for each calibration curve are tabulated.

Compound	Linearity Range (μg/mL)	LLOD (μg/mL)	LLOQ (μg/mL)	Linear Regression Equation	R^2^
CBDVA	50.0–333.3	18.1	54.8	y = 2.2 × 10^7^ × + 8.9 × 10^5^	0.999
CBDA	50.0–333.3	22.8	69.2	y = 2.6 × 10^7^ × + 9.3 × 10^5^	0.998
CBGA	50.0–333.3	31.1	94.2	y = 2.7 × 10^7^ × + 1.1 × 10^6^	0.997
THCVA	50.0–333.3	43.6	132.2	y = 2.1 × 10^7^ × + 8.9 × 10^5^	0.993
THCA	50.0–333.3	22.0	66.7	y = 1.9 × 10^7^ × + 9.0 × 10^5^	0.998
CBCA	50.0–333.3	30.5	92.3	y = 2.5 × 10^7^ × + 9.9 × 10^5^	0.997
CBD	100.0–666.7	53.1	161.0	y = 8.3 × 10^6^ × + 3.1 × 10^5^	0.996
CBN	5.0–500.0	16.6	50.3	y = 2.3 × 10^7^ × + 8.4 × 10^4^	0.999
Δ^9^-THC	100.0–666.7	48.9	148.3	y = 6.8 × 10^6^ × + 1.1 × 10^5^	0.997

**Table 3:** Experimental conditions of the supercritical CO_2_ extraction experiments (extraction feed, extraction time, co-solvent), along with the corresponding mean yields, total cannabinoids (CNBs, mg/g), total phenolic content (TPC, mg GAE/g), total flavonoid content (TFC, mg QE/g), and DPPH IC_50_ values (mg/mL). Results are expressed per gram of dry extract, whereas values in parentheses refer to mg/g of plant material (*n* = 2).

Νο.	Feed (g)	Time (h)	Co-Solvent (mL)	Yield% (*w*/*w*)	Total CNBs ^1^	TPC	TFC	DPPH IC_50_
1	30.7	4	-	0.15	-	113.62 (0.17)	53.60 (0.08)	5.61
2	10.0	4	-	0.31	-	163.44 (0.51)	72.34 (0.22)	4.98
3	4.7	4	-	2.74	737.28 (20.17)	227.23 (6.23)	82.38 (2.26)	2.03
4	5.0	4	15 (1:3)	8.38	414.98 (34.78)	191.44 (16.04)	167.69 (14.05)	2.92
5	5.1	4	30 (1:6)	6.04	399.48 (24.13)	205.70 (12.42)	157.23 (9.50)	4.71
6	5.0	4	25 (1:5)	7.59	413.80 (31.39)	230.74 (17.51)	158.54 (12.03)	4.24
7 ^1^	5.0	12	23.5 (1:5)	15.42	446.29 (68.84)	208.99 (32.23)	181.62 (28.01)	3.37
7.1		(0–4)		7.82	461.39 (36.08)	194.61 (15.22)	181.29 (14.18)	3.49
7.2		(4–8)		6.58	416.36 (27.41)	228.29 (15.02)	182.82 (12.03)	3.42
7.3		(8–12)		1.02	523.59 (5.35)	194.61 (1.99)	176.47 (1.80)	2.16

^1^ Values represent the weighted mean of the 7.1, 7.2, and 7.3 extracts and the cumulative value of the recovery.

**Table 4:** Cannabinoid content in the scCO_2_ cannabis extracts determined via LC-MS. Results are expressed as the mean mg/g of dry extract (*n* = 2), whereas values in parentheses refer to mg/g of plant material extracted.

No.	CBD	Δ^9^-THC	CBDA	CBDVA	CBGA	THCA	CBCA	Total CBD ^1^	Total THC ^2^	Total CNBs
3	478.52 (13.09)	18.04 (0.49)	236.20 (6.46)	DNQ	4.52 (0.12)	DNQ	DNQ	685.67 (18.76)	18.04 (0.49)	737.28 (20.17)
4	53.49 (4.48)	DNQ	273.21 (22.90)	29.27 (2.45)	8.59 (0.72)	21.01 (1.76)	29.43 (2.47)	293.09 (24.57)	18.43 (1.54)	414.98 (34.78)
5	64.27 (3.88)	DNQ	257.96 (15.58)	22.32 (1.35)	7.40 (0.45)	18.09 (1.09)	29.44 (1.78)	290.50 (17.55)	15.87 (0.96)	399.48 (24.13)
6	91.54 (6.94)	DNQ	254.38 (19.30)	19.87 (1.51)	9.23 (0.70)	15.25 (1.16)	23.54 (1.79)	314.63 (23.87)	13.37 (1.01)	413.80 (31.39)
7 ^3^	121.12 (18.68)	0.88 (0.14)	267.41 (41.25)	19.05 (2.94)	4.03 (0.62)	12.46 (1.92)	21.34 (3.29)	355.63 (54.86)	11.82 (1.82)	446.29 (68.84)
7.1	119.81 (9.37)	DNQ	277.25 (21.68)	19.66 (1.54)	7.95 (0.62)	13.87 (1.08)	22.85 (1.79)	362.96 (28.38)	12.16 (0.95)	461.39 (36.08)
7.2	112.51 (7.41)	DNQ	254.43 (16.75)	18.31 (1.21)	DNQ	11.01 (0.72)	20.11 (1.32)	335.64 (22.10)	9.65 (0.64)	416.36 (27.41)
7.3	186.60 (1.91)	13.35 (0.14)	275.66 (2.82)	19.14 (0.20)	DNQ	11.09 (0.11)	17.74 (0.18)	428.36 (4.38)	23.07 (0.24)	523.59 (5.35)

DNQ: detected, not quantified (value << LLOQ); CBN is not shown, although detected in all samples, it was not quantifiable (DNQ); ^1^ Total CBD calculated using the formula: Total CBD = CBD + [(CBDA × (MW_CBD_/MW_CBDA_)]; ^2^ Total THC calculated using the formula: Total THC = THC + [(THCA × (MW_THC_/MW_THCA_)]; ^3^ The values represent the weighted mean of the 7.1, 7.2, and 7.3 extracts and the cumulative value recovery.

## Data Availability

The original contributions presented in this study are included in the article/Supplementary Materials. Further inquiries can be directed to the corresponding authors.

## Supplementary Materials

The following supporting information can be downloaded at: https://www.mdpi.com/article/10.3390/antiox14070777/s1, Figure S1: Stacked LC-MS total ion chromatograms of the reference analytical standards used (in order: CBDVA, CBD, CBDA, CBGA, CBN, Δ^9^-THC, THCVA, Δ^8^-THC, THCA and CBCA) obtained in negative ionization mode; Figure S2: Chemical composition of the supercritical CO_2_ extracts studied. Bar graph depicts the mean values of total phenolic content (TPC, mg GAE/g of dry extract), total flavonoid content (TFC, mg QE/g of dry extract), and total cannabinoids (CNBs, mg/g of dry extract); error bars represent the SD (for TPC and TFC) and 95% CI (for total CNBs); Figure S3: Recovery of phenolic compounds, flavonoids, and cannabinoids from hemp flowers via supercritical CO_2_ extraction. Bar graph depicts the mean values of total phenolic content (TPC, mg GAE/g of plant material), total flavonoid content (TFC, mg QE/g of plant material), and total cannabinoids (CNBs, mg/g of plant material); error bars represent the SD (for TPC and TFC) and 95% CI (for total CNBs); Table S1: Cannabinoid acid content of cannabis flowers (THC dominant) upon decarboxylation at 145 °C and different time durations for the spike study. Values are expressed as compound mg per g of plant material; Table S2: Accuracy of the LC-MS method, evaluated by determining the recovery (*n* = 3) of each cannabinoid at three concentration levels over three consecutive days; Table S3: Precision of the LC-MS method, assessed by evaluating the intra-day (*n* = 3) and inter-day (*n* = 9) precision of each cannabinoid at three concentration levels over three consecutive days; Table S4: Cannabinoid content of hemp flowers, determined via UAE and LC-MS analysis (*n* = 11), expressed as mg/g of plant material.
