# Supplementary material for: Development of a Validated LC-MS Method for the Determination of Cannabinoids and Evaluation of Supercritical CO2 vs. Ultrasound-Assisted Extraction in Cannabis sativa L. (Kompolti cv.)
Source: Antioxidants (Basel). 2025 Jun 24;14(7):777. doi: 10.3390/antiox14070777 (PMC12291681; doi:10.3390/antiox14070777)
Supplement: Supplementary file 1 [file antioxidants-14-00777-s001.zip › antioxidants-3680532-supplementary.pdf]

Supplementary Material

Development of a Validated LC-MS Method for the Determination of Cannabinoids and Evaluation of Supercritical CO_2 vs. Ultrasound-Assisted Extraction in *Cannabis sativa* L. (Kompolti cv.)

Vasileios A. Ioannidis [1,2], Varvara Sygouni [2,3,*], Sotirios Giannopoulos [1], Konstantinos Sotirianos [3], Theophilos Ioannides [2], Christakis A. Paraskeva [2,3] and Fotini N. Lamari [1,*]

1 Department of Pharmacy, University of Patras, GR-26504 Patras, Greece; ioannidis.v@ac.upatras.gr (V.A.I.); up1088246@ac.upatras.gr (S.G.)
2 Foundation for Research and Technology, Hellas, Institute of Chemical Engineering Sciences, FORTH/ICE-HT, GR-26504 Patras, Greece; theo@iceht.forth.gr (T.I.); takisp@chemeng.upatras.gr (C.A.P.)
3 Department of Chemical Engineering, University of Patras, GR-26504 Patras, Greece; konstantinos.sotirianos@gmail.com
* Correspondence: sygouni@upatras.gr (V.S.); flam@upatras.gr (F.N.L.); Tel.: +30-2610-962335 (F.N.L.)

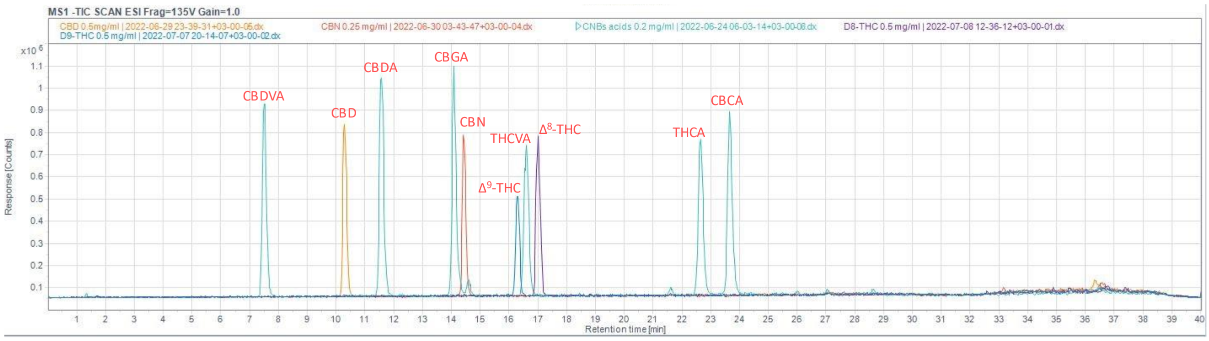

Figure S1: Stacked LC-MS total ion chromatograms of the reference analytical standards used (in order: CBDVA, CBD, CBDA, CBGA, CBN, Δ9-THC, THCVA, Δ8-THC, THCA and CBCA) obtained in negative ionization mode.

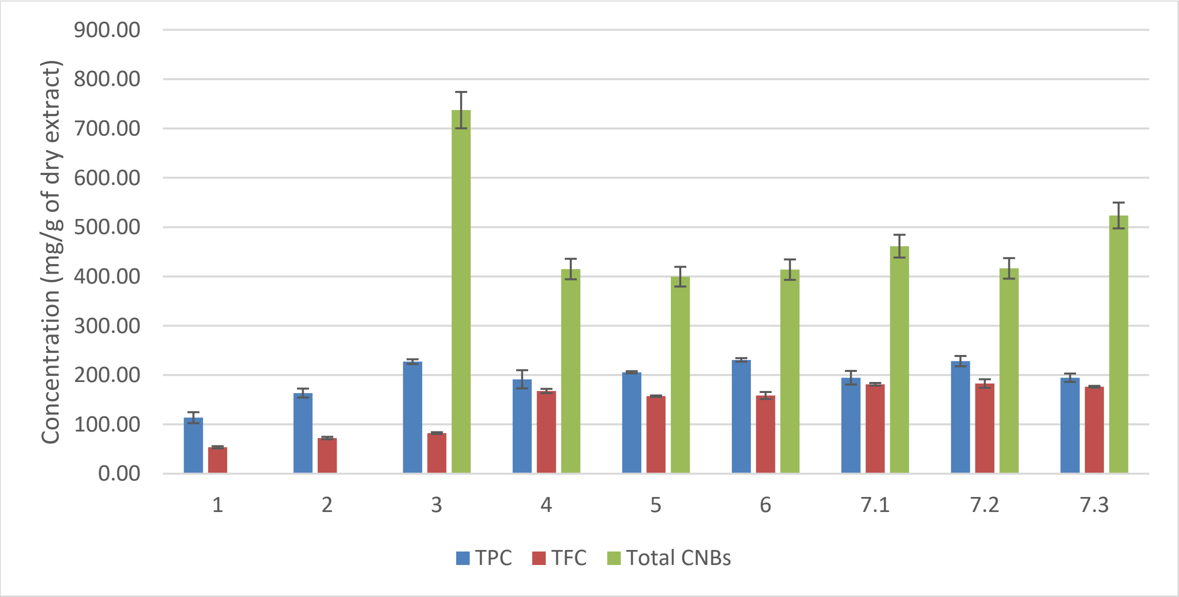

Figure S2: Chemical composition of the supercritical CO_2 extracts studied. Bar graph depicts the mean values of total phenolic content (TPC, mg GAE/g of dry extract), total flavonoid content (TFC, mg QE/g of dry extract), and

total cannabinoids (CNBs, mg/g of dry extract); error bars represent the SD (for TPC and TFC) and 95% CI (for total CNBs).

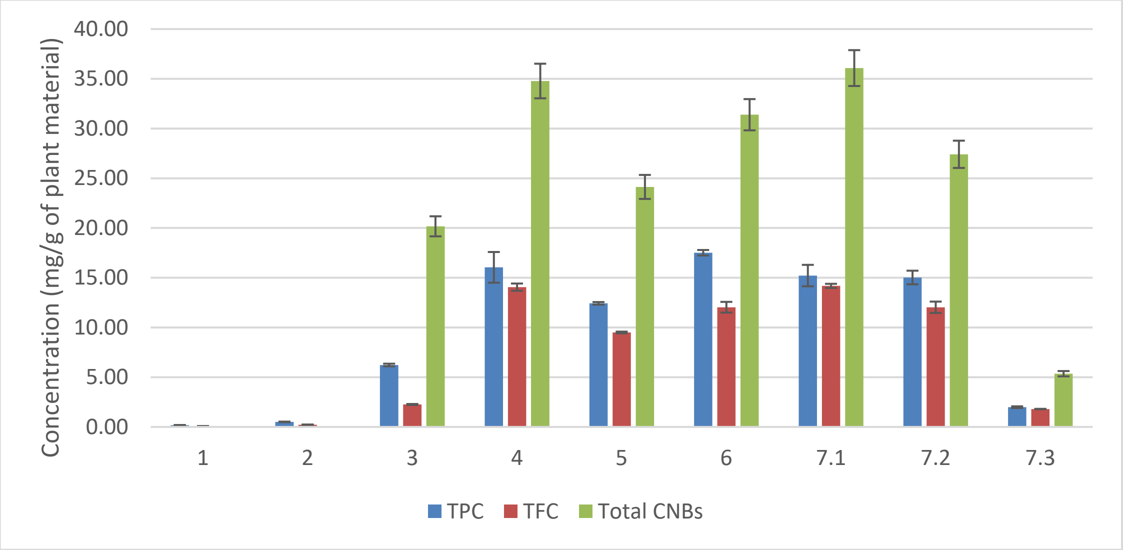

Figure S3: Recovery of phenolic compounds, flavonoids, and cannabinoids from hemp flowers via supercritical CO_2 extraction. Bar graph depicts the mean values of total phenolic content (TPC, mg GAE/g of plant material), total flavonoid content (TFC, mg QE/g of plant material), and total cannabinoids (CNBs, mg/g of plant material); error bars represent the SD (for TPC and TFC) and 95% CI (for total CNBs).

Table S1: Cannabinoid acid content of cannabis flowers (THC dominant) upon decarboxylation at 145 °C and different time durations for the spike study. Values are expressed as compound mg per g of plant material.

Time (min)	CBDA	THCA	CBCA	CBGA	CBDVA	THCVA
0	2.29	28.97	0.79	2.40	ND	DNQ
30	ND	DNQ	ND	ND	ND	ND
45	ND	ND	ND	ND	ND	ND
60	ND	ND	ND	ND	ND	ND

DNQ: detected, not quantified, ND: not detected

Table S2: Accuracy of the LC-MS method, evaluated by determining the recovery (n = 3) of each cannabinoid at three concentration levels over three consecutive days.

	Concentration level	Day 1 (n = 3) Amount found (µg/mL)	Amount added (µg/mL)	Recovery (%)	Day 2 (n = 3) Amount found (µg/mL	Amount added (µg/mL)	Recovery (%)	Day 3 (n = 3) Amount found (µg/mL)	Amount added (µg/mL)	Recovery (%)
CBDVA	Upper	279.9	300.0	93.30	305.6	300.0	101.89	306.1	300.0	102.04
	Mid	210.2	200.0	105.08	200.9	200.0	100.45	217.1	200.0	108.54
	Lower	140.4	125.0	112.32	126.0	125.0	100.80	137.3	125.0	109.87
CBDA	Upper	292.0	300.0	97.33	304.3	300.0	101.45	305.4	300.0	101.79
	Mid	208.9	200.0	104.47	204.3	200.0	102.16	206.0	200.0	102.99
	Lower	136.4	125.0	109.12	127.3	125.0	101.88	133.9	125.0	107.11
CBGA	Upper	299.3	300.0	99.77	298.0	300.0	99.34	304.1	300.0	101.38
	Mid	202.6	200.0	101.32	200.4	200.0	100.19	204.3	200.0	102.16
	Lower	120.9	125.0	96.76	122.3	125.0	97.90	129.5	125.0	103.64
THCVA	Upper	304.0	300.0	101.35	301.3	300.0	100.42	306.1	300.0	102.03
	Mid	208.8	200.0	104.41	201.8	200.0	100.92	215.8	200.0	107.89
	Lower	133.4	125.0	106.74	130.8	125.0	104.62	136.1	125.0	108.85
THCA	Upper	281.4	300.0	93.79	300.5	300.0	100.16	302.2	300.0	100.75
	Mid	214.2	200.0	107.14	207.9	200.0	103.93	213.1	200.0	106.54
	Lower	147.5	125.0	118.03	137.7	125.0	110.13	145.8	125.0	116.61
CBCA	Upper	264.3	300.0	88.10	268.4	300.0	89.48	270.9	300.0	90.30
	Mid	200.5	200.0	100.27	186.2	200.0	93.12	205.7	200.0	102.86
	Lower	124.8	125.0	99.81	128.5	125.0	102.79	131.0	125.0	104.84
CBD	Upper	467.6	600.0	77.93	483.7	600.0	80.61	474.4	600.0	79.06
	Mid	280.7	300.0	93.57	292.0	300.0	97.32	294.9	300.0	98.31
	Lower	161.7	200.0	80.84	178.4	200.0	89.21	175.4	200.0	87.71
CBN	Upper	427.9	400.0	106.98	392.1	400.0	98.03	381.5	400.0	95.39
	Mid	252.4	250.0	100.96	232.2	250.0	92.87	246.3	250.0	98.51
	Lower	103.6	100.0	103.61	96.2	100.0	96.20	97.0	100.0	97.00
Δ^9-THC	Upper	570.4	600.0	95.07	518.7	600.0	86.50	609.5	600.0	101.59
	Mid	327.8	300.0	109.27	301.8	300.0	100.60	317.4	300.0	105.79
	Lower	189.8	200.0	94.90	180.3	200.0	90.15	191.5	200.0	95.75

Table S3: Precision of the LC-MS method, assessed by evaluating the intra-day (n = 3) and inter-day (n = 9) precision of each cannabinoid at three concentration levels over three consecutive days.

	Concentration Level	Day 1 (n = 3)			Day 2 (n = 3)			Day 3 (n = 3)			Inter-day (n = 9)		
		Conc. (µg/mL)	SD	RSD	Conc. (µg/mL)	SD	RSD	Conc. (µg/mL))	SD	RSD	Conc. (µg/mL)	SD	RSD
CBDVA	Upper	279.9	0.009	3.08	305.6	0.001	0.40	306.1	0.005	1.56	297.2	0.014	4.68
	Mid	210.2	0.004	2.03	200.9	0.007	3.36	217.1	0.003	1.40	209.4	0.008	3.93
	Lower	140.4	0.008	5.52	126.0	0.002	1.89	137.3	0.001	0.79	134.6	0.008	5.75
CBDA	Upper	292.0	0.004	1.21	304.3	0.004	1.24	305.4	0.003	0.91	300.6	0.007	2.36
	Mid	208.9	0.002	0.84	204.3	0.001	0.33	206.0	0.002	1.07	206.4	0.002	1.21
	Lower	136.4	0.007	4.82	127.3	0.001	0.87	133.9	0.005	4.09	132.5	0.006	4.46
CBGA	Upper	299.3	0.003	0.89	298.0	0.004	1.50	304.1	0.002	0.51	300.5	0.004	1.29
	Mid	202.6	0.003	1.46	200.4	0.005	2.33	204.3	0.003	1.30	202.4	0.004	1.74
	Lower	120.9	0.003	2.35	122.3	0.002	1.68	129.5	0.004	3.23	124.3	0.005	3.89
THCVA	Upper	304.0	0.006	2.01	301.3	0.001	0.48	306.1	0.002	0.76	303.8	0.004	1.30
	Mid	208.8	0.002	0.95	201.8	0.001	0.36	215.8	0.003	1.53	208.8	0.006	3.04
	Lower	133.4	0.000	0.29	130.8	0.003	2.55	136.1	0.004	2.62	133.4	0.003	2.51
THCA	Upper	281.4	0.010	3.41	300.5	0.001	0.37	302.2	0.004	1.31	294.7	0.011	3.84
	Mid	214.2	0.007	3.43	207.9	0.001	0.64	213.1	0.007	3.44	211.7	0.006	2.84
	Lower	147.5	0.004	2.80	137.7	0.003	1.83	145.8	0.003	2.09	143.4	0.006	3.89
CBCA	Upper	264.3	0.003	1.21	268.4	0.005	1.90	270.9	0.002	0.87	267.9	0.004	1.67
	Mid	200.5	0.003	1.54	186.2	0.001	0.76	205.7	0.005	2.61	197.5	0.009	4.71
	Lower	124.8	0.001	0.54	128.5	0.004	2.73	131.0	0.000	0.21	128.1	0.003	2.55
CBD	Upper	467.6	0.010	2.23	483.7	0.002	0.45	474.4	0.019	3.92	475.2	0.013	2.69
	Mid	280.7	0.012	4.23	292.0	0.011	3.69	294.9	0.015	5.01	289.2	0.013	4.39
	Lower	161.7	0.006	4.02	178.4	0.007	4.12	175.4	0.002	1.11	171.8	0.009	5.36
CBN	Upper	427.9	0.009	2.02	392.1	0.010	2.51	381.5	0.009	2.46	400.5	0.023	5.63
	Mid	252.4	0.010	3.94	232.2	0.003	1.12	246.3	0.007	2.98	243.6	0.011	4.51
	Lower	103.6	0.003	2.61	96.2	0.002	2.18	97.0	0.002	1.66	98.9	0.004	4.04
Δ^9-THC	Upper	570.4	0.029	5.14	518.7	0.013	2.52	609.5	0.023	3.72	566.2	0.044	7.78
	Mid	327.8	0.002	0.51	301.8	0.007	2.40	317.4	0.003	0.96	315.7	0.012	3.81
	Lower	189.8	0.008	4.11	180.3	0.001	0.48	191.5	0.008	4.07	187.2	0.008	4.06

Table S4: Cannabinoids content of hemp flowers, determined via UAE and LC-MS analysis (n = 11), expressed as mg/g of plant material.

	CBD	THC	CBN	CBDA	THCA	CBCA	CBGA	CBDVA	THCVA	SUM	Total CBD
**1**	39.74	2.19	DNQ	38.38	DNQ	1.64	0.56	2.49	ND	85.00	73.40
**2**	40.87	2.05	DNQ	33.73	DNQ	1.57	0.61	2.51	ND	81.33	70.45
**3**	40.61	2.08	DNQ	35.34	DNQ	1.72	0.72	2.50	ND	82.96	71.61
**4**	45.64	2.12	DNQ	36.65	DNQ	1.50	0.64	2.48	ND	89.04	77.78
**5**	45.14	2.29	DNQ	38.86	DNQ	1.73	0.67	2.82	ND	91.50	79.22
**6**	40.24	2.05	DNQ	37.01	DNQ	1.69	0.57	2.55	ND	84.10	72.69
**7**	38.08	1.66	DNQ	34.22	DNQ	1.39	0.49	1.91	ND	77.75	68.09
**8**	38.93	1.73	DNQ	32.09	DNQ	1.49	0.67	1.94	ND	76.85	67.07
**9**	38.29	2.03	DNQ	31.52	DNQ	1.42	0.68	1.84	ND	75.78	65.93
**10**	40.99	1.98	DNQ	35.49	DNQ	1.54	0.65	1.97	ND	82.61	72.11
**11**	45.64	1.96	DNQ	38.58	DNQ	1.74	0.60	2.20	ND	90.71	79.48
**MEAN**	41.29	2.01	-	35.62	-	1.58	0.62	2.29	-	83.42	72.53
**SD**	2.73	0.17	-	2.43	-	0.12	0.06	0.31	-	5.15	4.47
**SEM**	0.82	0.05	-	0.73	-	0.04	0.02	0.09	-	1.55	1.35
**RSD**	6.61	8.67	-	6.81	-	7.63	9.81	13.73	-	6.18	6.16

DNQ: detected, not quantified (<LLOQ), ND: not detected
